# CRMAGE: CRISPR Optimized MAGE Recombineering

**DOI:** 10.1038/srep19452

**Published:** 2016-01-22

**Authors:** Carlotta Ronda, Lasse Ebdrup Pedersen, Morten O. A. Sommer, Alex Toftgaard Nielsen

**Affiliations:** 1 The Novo Nordisk Foundation Center for Biosustainability, Technical University of Denmark, Kogle Allé 6, 2970 Hørsholm, Denmark

## Abstract

A bottleneck in metabolic engineering and systems biology approaches is the lack of efficient genome engineering technologies. Here, we combine CRISPR/Cas9 and λ Red recombineering based MAGE technology (CRMAGE) to create a highly efficient and fast method for genome engineering of *Escherichia coli*. Using CRMAGE, the recombineering efficiency was between 96.5% and 99.7% for gene recoding of three genomic targets, compared to between 0.68% and 5.4% using traditional recombineering. For modulation of protein synthesis (small insertion/RBS substitution) the efficiency was increased from 6% to 70%. CRMAGE can be multiplexed and enables introduction of at least two mutations in a single round of recombineering with similar efficiencies. PAM-independent loci were targeted using degenerate codons, thereby making it possible to modify any site in the genome. CRMAGE is based on two plasmids that are assembled by a USER-cloning approach enabling quick and cost efficient gRNA replacement. CRMAGE furthermore utilizes CRISPR/Cas9 for efficient plasmid curing, thereby enabling multiple engineering rounds per day. To facilitate the design process, a web-based tool was developed to predict both the λ Red oligos and the gRNAs. The CRMAGE platform enables highly efficient and fast genome editing and may open up promising prospective for automation of genome-scale engineering.

*E. coli* is one of the most widely used model organisms for metabolic engineering for production of a wide range of biochemicals^1^. The use of systems biology, synthetic biology and evolutionary engineering has enabled an extensive portfolio of genetic tools and protocols for efficient, fast and cheap manipulation in order to make *E. coli* suitable for industrial applications. Homologous recombination (RecET)^2^, group II intron retrohoming^3^,^4^,^5^ and phage-derived recombinases (λ Red)^6^,^7^,^8^ have been applied to introduce modifications in the genome such as single point mutations or Knock-In/Out of genes. However in order to select for the recombinant clones, these methods rely on antibiotic markers, which have to be removed in order to introduce further modifications. Recycling of the selective marker can be achieved by using Cre-Lox recombinase^5^ or FLP flippase^4^ but both these enzymes leave scars in the genome. This limits the application for sequential allelic exchange since the scars will increase the risk of internal chromosomal rearrangements. In order to overcome the problem of selecting the recombinant strains and recycling of antibiotic markers, different methods of scarless counter-selection have been developed, including a SacB-based method^9^ and the use of the meganucleases such as Sce-I^10^,^11^. Despite the high efficiency of the counter-selection, the repeated engineering process becomes laborious and time consuming with multiple steps involved for the introduction of just a single modification. Additionally, the methods do not enable multiplexing since only few selective markers are available for combinatorial use.

The λ Red single/double-stranded DNA (ssDNA/dsDNA)-based mediated engineering is currently the only method that allows multiplexing in one step by direct electroporation of single stranded oligos or PCR products. For this reason it has been extensively used as editing tool and it has been employed for Multiplex Automated Genome Engineering (MAGE)^12^,^13^,^14^, a method that facilitates genome-scale engineering and barcoded genome profiling. It has recently been improved by co-operative oligonucleotide co-selection by leveraging selectable markers within 500 kb of multiple targets^15^. Moreover it has been demonstrated that it is possible to increase the number of recombinants by using mismatch repair mutants such as *mutS*^16^ and by using phosphorothioate linkages to protect the lagging-targeting strand in order to increase the half-life of the Oligos or PCR products and thus the probability of their incorporation during the replication^17^. The efficiency of short ssDNA/dsDNA oligonucleotide-mediated recombineering (including MAGE) is highest for short genome modifications, where around 6 to 20% can be achieved after single or multiple cycles respectively^13^,^18^, while larger modifications occur with significantly lower frequency (<1%)^13^,^17^. Therefore, PCR screening is always required^18^ making it challenging to identify the desired mutations that do not result in a clear phenotypic change.

CRISPR/Cas9 (clustered regularly interspaced short palindromic repeats and its associated protein, Cas9) system has recently proven to be a powerful tool for genome engineering in different organism, both Eukaryotes and Prokaryotes such as *E. coli*^19^,^20^,^21^ *Actinomycetal spp*^22^. *Streptomyces spp*.^23^,^24^, lactic acid bacteria^25^, *S.cerevisiae*^26^,^27^,^28^,^29^,^30^,^31^, higher plants^32^,^33^ *Bombyx mori*^34^, Drosophila^35^, Zebrafish^36^, human cell lines^37^,^38^ and CHO^39^,^40^,^41^ cells. The type II CRISPR-Cas system from *Streptococcus pyogenes* consists of the CRISPR- associated (Cas) protein, Cas9, a trans-activating CRISPR RNA (tracrRNA), and a programmable CRISPR targeting RNA (crRNA) that can be fused together to the tracrRNA in a synthetic sgRNA. Cas9 is a programmable nuclease that mediates blunt double-stranded break (DSB) at almost any target DNA locus were a PAM motif (protospacer-adjacent motif) is present at 3′ end^42^,^43^. In the case of *S. pyogenes* nuclease, the sgRNA scaffold can be programmed for a specific site by including 20 bp of the target locus at the 5′ position of the double guanine PAM motif (NGG) (20N-NGG). It is also possible to reprogram Cas9 by using tracrRNA and a synthetic array containing 30 bp of the target (5′ of NGG) embedded between two repeat regions that will be subsequently be processed in the mature crRNA^42^. In both cases the PAM motif is not included in the target sequence used for the sgRNA or crRNA array. CRISPR/Cas9 can be used to create a selective pressure during the recombineering procedure. The nuclease activity targets the loci that have not incorporated the desired mutation, where it induces double strand breaks (DSB) that have a significant fitness cost and may even result in cell death. In *E. coli*, the CRISPR-Cas9 system has been recently coupled to λ Red oligos recombineering in order to improve its efficiency^19^,^20^,^21^. Recently, Jiang *et al.*^19^ were able to reach high efficiency of recombineering using dsDNA oligos by expressing in succession the two systems. Pyne *et al.* have also recently demonstrated that the CRISPR/Cas9 system combined with λ Red is a promising strategy to facilitate scar-less large chromosomal gene replacement^20^ and multiple knock-outs and knock-ins of both small and large fragments^21^. However both of these methods rely mainly on dsDNA oligos, which always require all the λ Red proteins (exo, β and γ) and it is not suitable for a high throughput genome scale approach based on chip-based synthetized oligos^44^,^45^. Additionally, the system reported by Jiang *et al.*^19^ system did not shown very high efficiencies when dsDNA was co-transformed with gRNA expressing plasmid. Li *et al.*^46^, have also demonstrated the efficacy of using λ Red and CRISPR in metabolic engineering of *E. coli* for integrating the β-carotene synthetic pathway into the genome, and used the same method to optimize its production by modifying the methylerythriol-phosphate (MEP) pathway and the central metabolic pathways. So far, none of the established methods are compatible with automation, which could further increase engineering throughput.

Here, we aimed at generating a simple and highly effective tool, CRMAGE, which enables fast genome editing compatible with automation protocols. CRMAGE exploits intrinsic negative selection against the wild type of CRISPR/Cas9 in order to increase the MAGE performance for small genome modifications such as codon substitution or translation control elements. The system is based on two curable plasmids that encode optimized versions of both systems λ Red recombineering and CRISPR/Cas9. Since both MAGE and gRNA oligos are critical for the protocol, we additionally created a web based tool for automating and optimizing the oligo design. We furthermore demonstrated that degenerate codon usage can be used to expand the range of CRISPR targets, thus creating a powerful tool to modify any site in the genome by introducing different types of modifications such as standard knock-Out/In, point mutations and modulation of protein translation in a single step.

## Results

### Construction of the CRMAGE system

**Figure 1:**
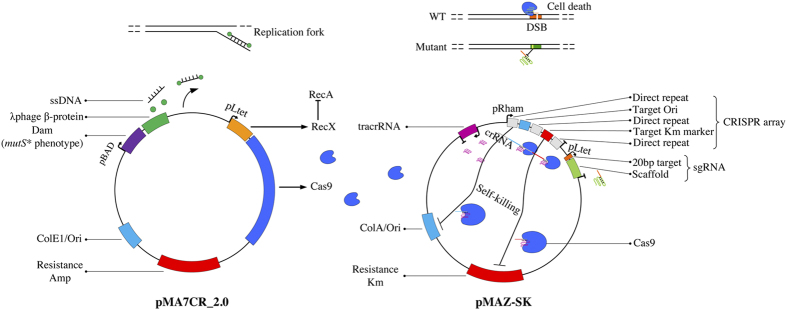
Schematic cartoon of CRMAGE system. CRMAGE consist of two plasmids, pMA7CR_2.0 expresses λ/RED β-protein and CRISPR/Cas9 protein that are inducible by L-Arabinose and aTetracyline respectively. The β-proteins are co-expressed with *dam*, which gives a *mutS* mutator phenotype, and *cas9* is expressed in a operon with *recX*, which blocks the repair of double strand breaks. The second plasmid (pMAZ-SK) contains an aTetracyline inducible sgRNA used for selection against the wild type sequence, as well as a self-eliminating circuit that targets its own backbone to enable plasmid recycling and sequential recombineering. Upon L-rhamnose induction (and aTetracycline for *cas9* induction), a tracrRNA that combines with two crRNAs, arranged in a natural CRISPR in order to target the origin (Ori) and the antibiotic marker (Kanamycin).

In order to increase the overall MAGE performance, CRMAGE technology exploits the ability of Cas9 to create DSB and thus kill cells that have not had the PAM sequence removed by the recombineering reaction. This method has been conceived to compact and optimize at the same time λ Red recombineering and CRISPR/Cas9 in one system ready to be applied for automation (Fig. 1). Therefore CRMAGE consist of just two plasmids, which are completely curable (Fig. 1). One plasmid (pMA7CR_2.0) expresses the λ Red β-protein and the CRISPR/Cas9 protein, which are inducible by L-arabinose and anhydrotetracyline (aTetracycline) respectively. The second vector is a recycling plasmid (pMAZ-SK) that contains an aTetracyline inducible sgRNA used for the negative selection, as well as a “self-destruction” gRNA cassette that targets the vectors own backbone in order to enable plasmid recycling and sequential recombineering steps (Fig. 1). The self-killing system consists on a tracrRNA that combines with two crRNAs, arranged in a natural CRISPR array (30 bp repeat −30 bp target −30 bp repeat −30 bp target etc.), to target the pCOLA plasmid origin (Ori) and the kanamycin antibiotic marker upon induction with L-rhamnose and aTetracycline. The λ Red has been coupled to a transient *mutS* phenotype as detailed below, in order to optimize the recombineering step by having a transient deficient repair system. We have also attempted to increase the efficiency of the negative selection of CRISPR/Cas9 by coupling it with a transient *recA*^-^ phenotype in order to promote cell death due to the inability to repair DSB as described below.

### CRISPR/Cas9 Killing efficiency and estimation of fitness advantage for protocol optimization

**Figure 2:**
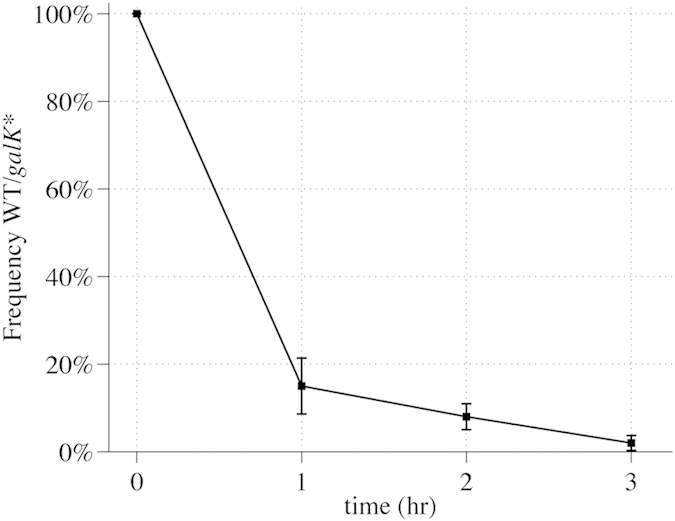
Wild type killing efficiency. The graph represents the killing of a wild type strain compared to a *galK* mutant over time. For each time point the cells were plated and the ratio between WT/galk* was calculated.

In order to investigate the efficacy of the CRISPR/Cas9 negative selection and to estimate the selection time required for the CRMAGE protocol we first determined the CRISPR/Cas9 killing rate and the chance of recombinants take-over using a gRNA targeting the metabolic gene encoding the galactokinase (*galK*). The *galK* gene was used as a target because loss of function of this gene is easy to screen for using MacConkey plates, where colonies capable of utilizing for example galactose will turn purple. The CRMAGE system (pMA7CR_2.0 and pMAZ-SK::galK2) was transformed into both the WT MG1655 strain and into an MG1655 strain harboring the *galK* mutation that confers CRISPR immunity. Using traditional recombineering, we constructed a *galK* mutant carrying a stop codon in position 38 (TAC > TAG), which causes truncation and loss of function of the galactokinase (Table 1 and Fig. S1). By inducing the CRMAGE system targeting the wild type *galK* sequence in both backgrounds, the survival of each strain was followed over time. The two strains were inoculated with the same amount of cells in two different flasks. Cells were grown exponentially to OD 0.4–0.5, after which the CRMAGE system was induced. Cells were plated before induction and each hour after induction and the killing rate was determined as the ratio of WT cells over the galk* mutant. Interestingly an almost complete killing of all WT cells was achieved only two hours after induction and almost no viable cells were detected after three hours (Fig. 2). Only few cells survived, potentially because the CRISPR system may be susceptible to escapers. Indeed only a single point mutation in the PAM motif or in the seed region (8 nucleotides upstream the PAM) can create resistance and thereby eliminating the killing/selection activity. Additionally, a competition experiment with the two strains mixed was also performed and resulted in identical data.

### RecA and *mutS* transient mutants contribution in CRMAGE optimization

**Figure 3:**
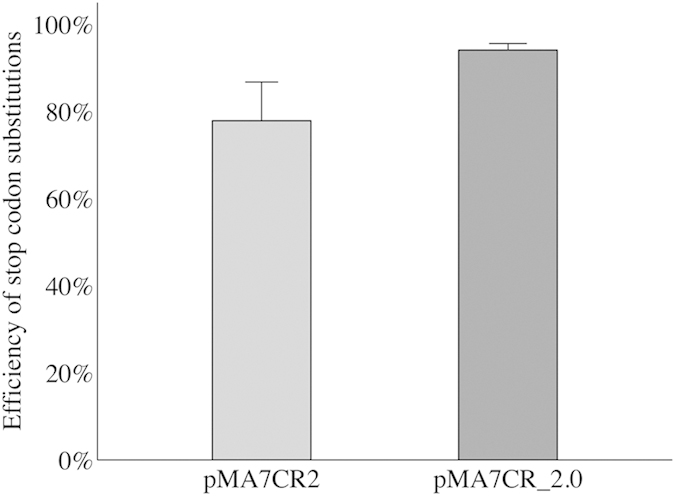
Contribution of RecX in CRMAGE efficiency. The graph shows the effect of *recX* expression on the CRISPR/Cas9 killing activity after λ Red recombineering. In pMA7CR_2.0 (represented by the dark grey bar on the right) *cas9* was expressed in a synthetic operon together with *recX*. In pMA7CR2, *cas9* was expressed alone (represented by light grey bar on the left). The presence of *recX* positively contributes to the negative selection (dark grey bar on the right) with p-value < 0.1 according to a T-test analysis.

In order to increase the efficiency of CRMAGE, we aimed at improving the efficiency of both the λ Red recombineering and the CRISPR/Cas9 killing. Costantino *et al.* has proven that defects in the mismatch repair system can enhance the efficiency of recombineering more than 100-fold^16^. We therefore created a transient *mutS*^*-*^ phenotype by overexpressing the Dam methyltransferase, since this has been documented to result in a mutator (*mutS*^−^) phenotype^47^,^48^,^49^. The *Dam* gene was expressed in an L-arabinose inducible synthetic operon together with the β-protein in order to obtain a transient mutator strain that can be induced only during the recombineering stage, thereby minimizing the creation of unwanted mutations. To optimize the negative selection of the CRISPR/Cas9 system, both *Cas9* and *recX* were expressed in a synthetic operon under control of the pLTet promoter. It has previously been shown that overexpression of RecX inhibits RecA activity^50^,^51^, one of the major components of the DNA repair system. Since *RecA* mutants are not capable of repairing DSBs (Double Strand Break), we wished to investigate if overexpression of the inhibitor (RecX) would enhance the CRMAGE negative selection. After one CRMAGE cycle using a galK MAGE oligo we observed that *recX* overexpression most likely has a smaller positive impact on CRMAGE efficiency (p-value <0.1) (Fig. 3), and we therefore decided to keep overexpression of *recX* in the CRMAGE system.

### Use of CRMAGE to engineer PAM-dependent and independent genomic targets

**Figure 4:**
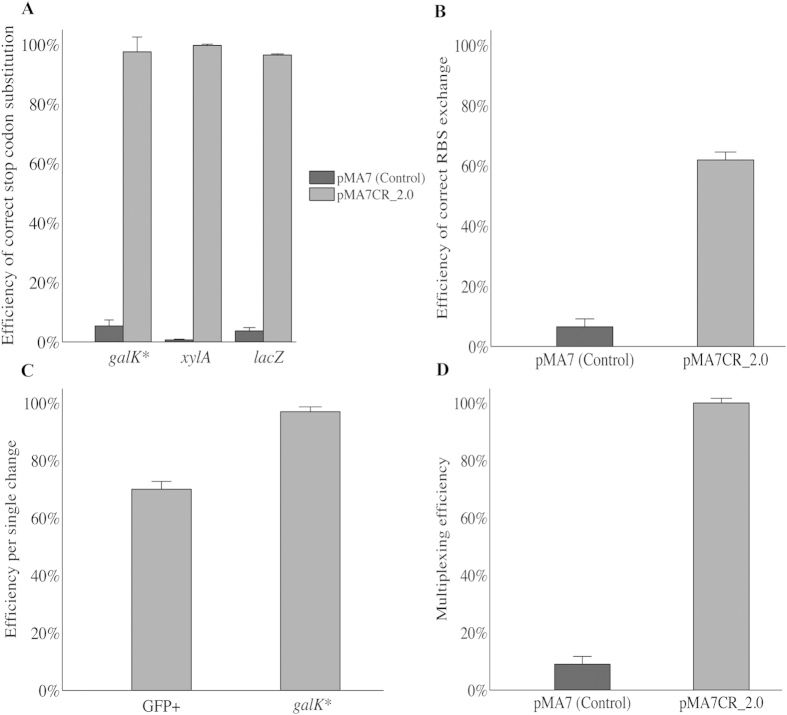
Efficiency of CRMAGE. Panel (**A**,**B**) show the efficiency of CRMAGE for gene recoding using pMA7CR_2.0 compared to the pMA7 control using only λ Red. (**A**) The efficiency of CRMAGE for introducing codon substitution stop codons in *xylA* and *lacZ*. Additionally, a stop codon was introduced in galk* linked to a secondary silent codon substitution used by CRMAGE as counter selection. (**B**) The efficiency of substituting a weak RBS in front of GFP with a strong one (TCCTCC > AGGAAG) while disrupting the PAM sequence used for negative selection. Panel (**C–D**) shows the efficiency of multiplex CRMAGE. (**C**) The efficiency of a single round of CRMAGE for simultaneously introducing the two mutations mentioned above (the RBS exchange on the left and stop codon substitution on the right). (**D**) CRMAGE multiplexing efficiency (pMA7CR_2.0) of the two mutations compared to the control (pMA7) using only λ Red. The data result from the analysis of 15 to 40 positive clones per each mutation that were re-streaked and tested if they were carrying also the second modification.

After estimating the optimal killing window for CRMAGE, we wanted to test if the system could be used to target loci in the genome that are not directly linked to a PAM sequence. As proof of concept we decided to create a *galK* knockout by changing one of the initial codons of the gene, which is not directly linked to a PAM site, into a stop codon (TAC > TAG) (Table 1). To do this, we identified a PAM site in the closest proximity (within the 70 bp coverage of the MAGE oligo) and we introduced a secondary silent mutation that disrupts the nearby PAM sequence while coding for the same amino acid (ACC > ACA) (Table 1). After a single round of CRMAGE, we found that about 98% (Fig. 4a) of the population had incorporated the desired mutation in *galK*. Using traditional MAGE only 5% of the population had the specific mutation incorporated (Fig. 4a), which is consistent with previous studies^18^. All mutations were confirmed by sequencing. This makes it theoretically possible to utilize the CRMAGE system to target any site in the genome with a very high efficiency thus further expanding the ability to investigate codon usage and re-coding of the essential genes, as it has been previously attempted using MAGE alone^52^. In order to investigate the robustness of the CRMAGE system, we tested two additional metabolic targets, *xylA* and *lacZ*. Using CRMAGE, a stop codon was introduced in both genes (Table 1 and Fig. S1), and the efficiency was assessed by plating on MacConkey plates. After a single round of CRMAGE, an efficiency of 99.7% and 96.5% was reached for *xylA* and *lacZ* respectively (Fig. 4a). Using traditional recombineering, only 0.6% efficiency for *xylA* and 3.6% for *lacZ* could be achieved, thus demonstrating the strength of CRMAGE.

### Use of CRMAGE for small insertions and modulation of protein synthesis

The high efficiency of the CRMAGE system for achieving single point mutations prompted us to test if CRMAGE would enable larger genome modifications, which typically result in very low frequencies when relying on MAGE alone^13^,^17^. Since it is often desirable to modulate expression level of certain proteins, we tested if CRMAGE could be used to modify regulatory elements such as RBS sequences. A strain harboring a genome integrated GFP with a weak RBS was used. We then attempted to introduce an RBS variant (TCCTCC > AGGAAG) (Table 1 and Fig. S1) predicted to have significantly higher expression levels, thus resulting in higher fluorescence levels (GFP^+^). Using the CRMAGE system, an efficiency of 62% was achieved (Fig. 4b), while only 6% of the population had introduced the RBS modification when relying on MAGE alone (Fig. 4b). All insertions were confirmed by sequencing. This demonstrates that it is feasible to generate libraries of different regulatory elements with high efficiency using CRMAGE.

### Use of CRMAGE for multiplexing

Given the high efficiency of introducing both single point mutations as well as a larger RBS modification, we decided to test if introduction of multiple mutations could be achieved in a single round of CRMAGE. In a wild type background, we therefore co-selected for the introduction of both the replacement of the RBS sequence in front of GFP and the introduction of a stop-codon in *galK.* The two different MAGE oligos were co-transformed together with two different gRNAs for CRISPR/Cas9 negative selection. The final population was plated on both MacConkey and LB plates and the efficiency was determined for each individual mutation (Fig. 4c). In randomly picked colonies, 98% were found to carry the galK* mutation and 70% carried the RBS modification (Fig. 4c). Subsequently, recombinant clones for each mutation (GFP^+^ and galk*) were tested to determine if they also carried the second mutation. All the tested colonies (40 out of 40) showing strong GFP fluorescence after CRMAGE also resulted in white colonies when plated on MacConkey plates, thus proving that they also incorporated the galK* mutation. Similarly, all the colonies tested (15 out of 15) carrying the galK* mutation also showed strong GFP expression, thus surprisingly resulting in a 100% efficiency of simultaneously introducing the double mutations (Fig. 4d). Using MAGE alone, only 5–6% of the colonies carried the galK* mutation, and of these only 10% carried showed strong GFP fluorescence (Fig. 4d). All alterations were confirmed by sequencing.

### Elimination of gRNA plasmid for subsequent rounds of CRMAGE

**Figure 5:**
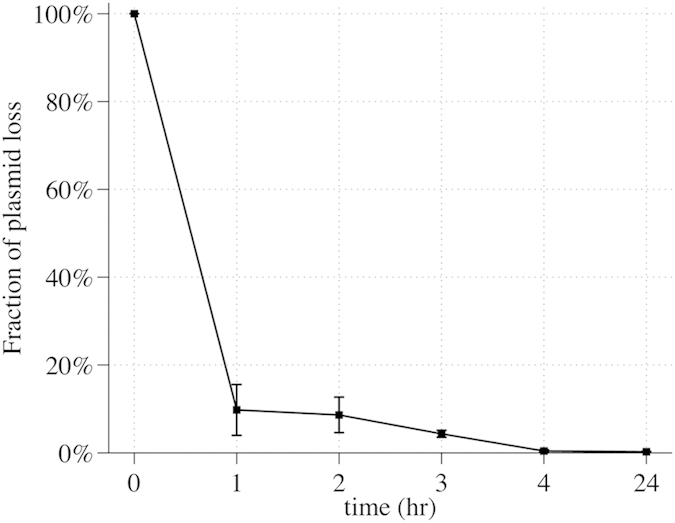
Self-eliminating proprieties of pMAZ-SK plasmid used for negative selection. The graph shows the rate of plasmid curing over time. The population was induced with L-rhamnose (to induce self-killing crRNA) and aTetracycline (to induce Cas9 expression). Cells were plated on LB plates with and without kanamycin. Plasmid loss was calculated as the fraction of kanamycin resistant colonies compared to the total number of colonies.

In order to facilitate sequential recombineering cycles, it is beneficial if the plasmid required for the negative selection can be quickly cured, so that a new vector can be introduced for the next round of CRMAGE. For this reason we designed the plasmid carrying the gRNAs as a self-killing plasmid (pMAZ-SK) by exploiting CRISPR/Cas9 ability to cut the plasmid DNA. An L-rhamnose inducible CRISPR natural array encoding two pre-crRNAs that target the origin of the plasmid and the kanamycin antibiotic marker was therefore included in the vector. In order to create mature active crRNA, the tracrRNA was included on the plasmid under the control of a strong synthetic constitutive promoter. This makes it possible to induce plasmid digestion in order to cure the plasmid before starting a new round of CRMAGE. We initially determined the minimum amount of time required for successful curing of the plasmid by following the number of cells that retained the plasmid after L-rhamnose induction (Fig. 5). After just 2–3 hours of induction, 92–96% of the cells had lost the plasmid. Interestingly, a complete removal of the plasmid was not achieved even after overnight incubation, where 0.2% of the population still retained the plasmid (Fig. 5). However, for the practical use for CRMAGE, very efficient plasmid loss can be achieved even after a short incubation time, which will significantly speed up the engineering process. The effective plasmid loss induced by the expression of tracRNA and the CRISPR array that targets the plasmid origin and the kanamycin cassette demonstrates that the processing of the RNA is functional. Using the same principle, it will therefore also be possible to include gRNAs that target the pMA7CR_2.0 plasmid, thereby making it possible to remove both plasmids at the end of the final engineering cycle.

### CRMAGE gRNA design tool

**Figure 6:**
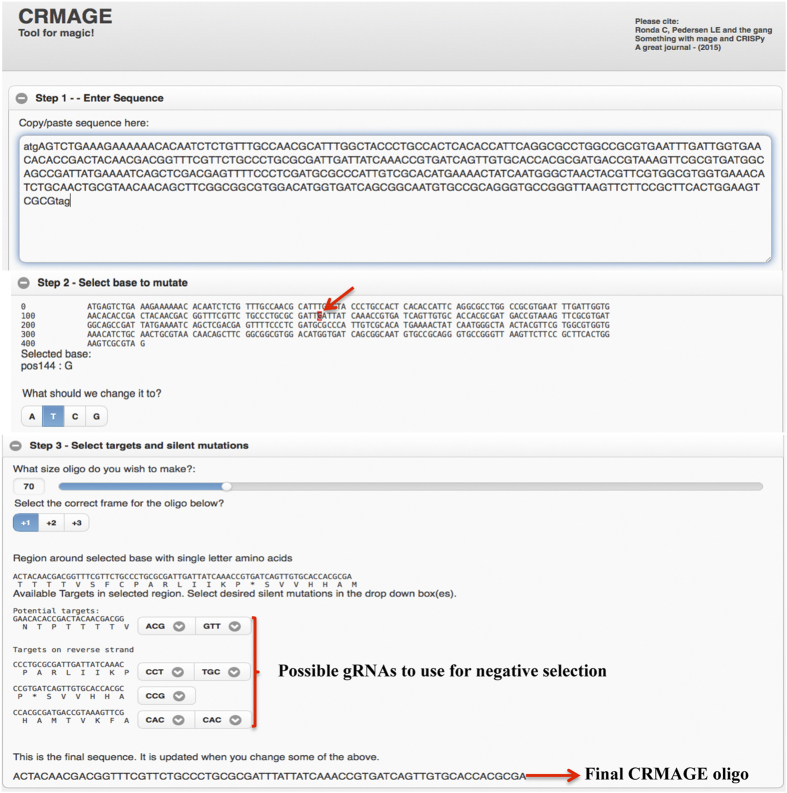
CRMAGE Web-Tool. The program guides the user through the necessary steps to create a CRMAGE mutated oligos, considering also the option of using a degenerate codon if the desired mutation does not contain a PAM itself. It consists of three major steps: (1) Enter (copy/paste) the wild type DNA sequence; (2) Select the base to mutate and which base to change to Step 3: Choose the Open Reading Frame and the size of the CRMAGE mutation oligo. A list of potential gRNA sites are shown, and the user can pick silent mutations to destroy the indicated gRNA PAM site (pointed by the red bracket). Finally the program outputs the CRMAGE oligo sequence (shown by the red arrow).

A number of tools are available online for identifying and designing gRNAs for various organisms. However, since CRMAGE enables the generation of specific mutations throughout the genome, it is necessary to both identify the optimal gRNA for counter selection, as well as the oligos required for MAGE. Furthermore, when the specific mutation is not located in close proximity of a PAM sequence, it is necessary to identify a nearby PAM sequence that can be included in the MAGE oligo design as described earlier. In order to automate the design of both sets of oligos, we have created a web based tool, CRMAGE – Web tool (available at http://staff.biosustain.dtu.dk/laeb/crmage/), which guides the user through the necessary steps to create a CRMAGE mutated oligos, considering also the option of using degenerate codons if the desired mutation is not located near a PAM sequence. It consists of 3 major steps which include: input of a DNA sequence to mutate, user based selection of the base(s) to mutate, and the program finally presents the user with available gRNAs near the desired mutation site as well as with options to mutate the PAM site of the gRNA to prevent it from selecting against the recombinants. The final output includes the gRNA as well as an SS-DNA oligo to use for recombineering that contains both the desired mutation and the one required for negative selection (Fig. 6). The highest efficiency will be achieved using an oligo targeting the lagging strand, and the user should account for this during the design.

## Discussion

CRMAGE is based on the combination of MAGE recombineering with the negative selection against the wild type sequence, which can be achieved using CRISPR/Cas9 targeted double strand breaks. In a single round of CRMAGE recombineering, we were able to achieve close to 98% efficiency for a single point mutation in *galK* versus 5% using traditional MAGE. The robustness of our system was demonstrated by introducing stop codons in two additional targets (lacZ and xylA) with efficiencies between 96.5% and 99.7%. For replacing a larger 6 bp RBS sequence, an average of 66% was achieved in two separate types of experiments, while only 6% efficiency was reached using traditional MAGE. Such high efficiency with ssDNA oligos and short homology arms (70 bp) has not been previously reported. Recently, Pyne *at al*.^20^, Jiang *et al.*^21^ and Li *et al.*^46^, have reported the use of CRISPR-Cas9 based technology to engineering *E. coli* with high efficiency. Pyne *at al*.^20^ achieved 39% for small insertions (8 bp) using dsDNA and 80% for small modifications with ssDNA and Jiang *et al.*^21^, could reach efficiencies in the range of 72% to 92% relying on long homology arms (300–400 pb), while focusing mainly on large modifications. The CRMAGE system additionally enables integration of multiple mutations with no apparent loss of efficiency, which is different from previous work where the multiplexing efficiency dropped considerably from 39–47% to 0.68-0.25% and from 72–92% to 48–78% in Pyne *et al.* and Jiang *et al.* systems respectively^20^,^21^. Jiang *et al.* combined gRNA expression cassette and donor DNA into a single vector rather than co-transforming a gRNA expressing plasmid and donor DNA. This design requires more laborious cloning steps, especially when targeting multiple sites. In the case of CRMAGE, we speculate that the reason for incomplete killing of the WT or rescue of the selection plasmid (pMAZ-SK) is the leakiness of the tetracycline and rhamnose promoters that may result in a selective pressure during the 5 hours of the whole CRMAGE protocol, which may result in few escapers that will not succumb to the negative selection.

It has previously been attempted to modulate chromosomal gene expression in one single step using PCR products^53^. However this work relied on phenotypic screening, and the method leaves FRT scars after the regulatory element is inserted. The presence of scars in the genome promotes genome rearrangement after several rounds of modifications thus making it difficult to further engineer the strain. With CRMAGE we have demonstrated that is possible to modulate the protein translation with relatively high efficiency within the population, which can be applied to any regulatory element that could be inserted to modulate either the gene expression or protein translation.

The CRMAGE system was furthermore designed to enable efficient recycling of the plasmid used for negative selection by targeting the Cas9 towards the origin and kanamycin cassette in the vector backbone itself. Controlled by a L-rhamnose inducible promoter, an almost complete loss (96%) of the plasmid was achieved after 2–3 hours of induction. This means that it is possible to proceed with subsequent rounds of CRMAGE without an intermediate screening for kanamycin sensitive clones. Using the same approach it will also be possible to remove the entire CRMAGE system in the last CRMAGE round, where the CRISPR/Cas9 killing activity can target origin and antibiotics markers in both plasmids thus resulting in a clean recombinant strain. The idea of generating a clean background strain and the possibility to recycle the plasmid containing the gRNA at the end of the engineering process has been considered by Jiang *et al.*^21^. In their work they have aimed at rescuing the plasmid harboring the gRNA for negative selection by induction with IPTG, and the plasmid carrying λ Red and Cas9 by growing the culture at 37 °C. However it was not possible to proceed with a subsequent round of recombineering before of 8 or 16 hours of IPTG induction. The use of the temperature sensitive origin used for plasmid encoding Cas9 furthermore increases the time required for one single round of recombineering since the growth rate of *E. coli* is significantly reduced at 30 °C. The method presented by Li *et al.*^46^ also require significant time per engineering cycle. Using CRMAGE it is possible to perform up to two cycles per day since more the 96% curing efficiency is reached after only 2–3 h of rhamnose induction, thus making almost unnecessary to plate the cells to cure the plasmid.

The timing of expression of the different components of the CRMAGE system has been carefully selected. The λ Red system is expressed first, and only after the cells have segregated, the CRISPR system is induced for negative selection. In a final step, the gRNA expressing plasmid can be cured and a new loop initiated. Constitutive expression of the gRNA and Cas9 may cause problems during the replication and the recombineering steps. This may explain the higher rate of escapers observed by Li *et al.*^46^.

For multiplexing of the CRMAGE targets, it is necessary to express multiple synthetic gRNAs that have significant stretches of sequence homology (typically 136 nucleotides). As discussed by Jiang *et al.*, the repeated sgRNAs may result in homologous recombination^21^. Here, we have shown that the presence of a constitutively transcribed tracRNA in the pMAZ-SK vector makes it possible to generate multiple gRNAs from a single short region carrying only 30 nucleotides per gRNA followed by a 30 nucleotide repeat between them. The synthesis and cloning of such an array of gRNAs (target-repeat-target-repeat etc.) is simple and it minimizes the risk of recombination in the vector. The expression of the pre-crRNA for multiplex CRMAGE may substitute the sgRNAs if put under control of the inducible pLtet promoter, making it easy to control the timing of the expression. The system will most optimally use aTetracycline induction for negative selection (when pLtet is used in front of the CRISPR array) and L-Rhamnose induction for curing the plasmid.

We furthermore demonstrate that degenerate codon usage can be used to expand the range of CRISPR targets, thus creating a powerful tool for targeting virtually any site in the genome and enabling single step engineering to create single point mutations as well as larger mutations, as demonstrated by the efficient RBS replacement for modulating protein translation. The same design can be applied for creating knock-ins or complete knock-outs, depending on the donor oligos. The web based CRMAGE web - tool has been engineered to facilitate the design of both the λ Red oligo required for the specific mutation as well as gRNA required for the negative selection and together with the convenient USER-cloning-assembly-based system we have developed, speed up the design and cloning process. Since CRMAGE enables the generation of multiple mutations in a single cycle and multiple cycles within a working day, it has the potential to significantly increase the daily strain engineering capacity. The increased efficiency furthermore opens up the possibility of automating genome-scale engineering.

## Materials and Methods

### Strains, Media and Reagents

*E. coli* K-12, MG1655 strain with genome integrated repressor from pZS4Int-tetR, was used to perform CRMAGE experiments and DH5α strain instead was used for cloning purposes. An MG1655 *galK* mutant carrying a stop codon in position 38 (TAC > TAG) was constructed using traditional recombineering with galK 2.2 CRMAGE oligo (Table S1). In an MG1655 strain harboring GFP integrated downstream of the *glmS* gene (the strain was a gift from Dr. S.I. Jensen), the RBS was exchanged with a weak one using traditional recombineering with oligo 35 (Table S1) and screened for loss of fluorescence. CRMAGE was performed using LB-Lennox (10 g/L tryptone (“Enzymatic digest from caseine”- Fluka Analytical), 5 g/L yeast extract (Fluka Analytical), 5 g/L NaCl) supplemented with 0.5 mM of MgSO_4_ (Sigma). All cultures were grown at 37 °C, 250 rpm shaking. For cloning Q5 high fidelity Polymerase (NEB), Fasta Digestion enzymes and Buffer from Fermantas/Thermo Scientific and USER enzymes from NebLab was used. All oligonucleotides and gblocks were synthesized by Integrated DNA Technologies (IDT; Coralville, IA) at the 25 nm scale using standard desalting.

### Plasmids constructions and CRMAGE plug and play BioBrick

The pMA7CR_2.0 (Amp^R^) has been constructed by cloning Cas9 and RecX in pMA7 plasmid^54^,^55^. *RecX* was first amplified from purified MG1655 genome using the primers 1 and 2 (Table S1) and cloned in pZ21MCS^50^ downstream of pLTet promoter using the restriction enzymes KpnI and BamHI. Then the expression cassette, gene + promoter (pLtet_recX), was amplified and cloned in pMA7^54^,^55^ with USER using primers 3, 4 and 5, 6 (Table S1) for the pMA7 backbone and RecX respectively. Eventually a synthetic operon was created by cloning Cas9 with a strong RBS (AAGGAGA) downstream pLTet_recX using primers 7, 8 to amplify the backbone with recX, and 9, 10 to amplify Cas9 directly from *S. pyogenes* M1 genome (ATCC collection) (Table S1). The plasmid pMACR_2.0 comes in combo with plasmid pZS4Int-tetR (CAM^R^)^56^ that is a single copy plasmid harboring TetR repressor necessary for the control of Cas9 and sgRNA expression. This plasmid can also be integrated in the genome in one single step using the helper plasmid pLDR8 [WM2269] (ATCC^®^ 77357) expressing the integrase. The self-killing gRNA plasmid (pMAZ-Self-Killing: pMAZ-SK) was designed using pCOLA-duet (Millipore) as backbone vector and it consists on two main parts. The first one contains the aTetracycline inducible synthetic gRNA use for negative selection and the second one constituted of two subparts for the self-killing (Fig. 1), the pre-crRNA array and the tracrRNA. The pre-crRNA array and the tracrRNA were ordered as synthetic oligos and gBlock from Life Technology and IDT respectively. The first one, named part1_selfkilling (Table S1) was designed to have the pre-crRNA array coding for two crRNA targeting the Origin (Ori) and the antibiotic marker (Kanamycin) of the pCOLA backbone, and it was cloned using the restriction enzymes *Nco*I and *Pac*I after the part containing the tracrRNA (part2_selfkilling) was cloned with USER using primers 11 to 14 (Table S1). In the part2_selfkilling component, there is the tracrRNA under a constitutive synthetic promoter and a synthetic terminator, both derived from igem.part.org (Table S1). Once the two major components of the self-killing plasmid were assembled in the pCOLA backbone, the synthetic gRNA for the negative selection was added using primers 17 to 20 (Table S1) with USER cloning^57^. Only the gRNA GFP4 (Table S1) was ordered as a gBlock (from IDT) to have it as a unit organized in: the aTetracycline inducible promoter (pLtet), 20 bp target sequence and the general scaffold that fuses part of the repeat region of the crRNA and the tracrRNA.

In order to change the target sequence in the synthetic gRNA we have designed a USER-cloning-assembly-based plug-and-play system, where it is only necessary to order two oligos to change the synthetic gRNA for the negative selection. This system relies on USER cloning^57^: the self-killing backbone was amplified using the universal primers 21, 22 (Table S1) containing the uracil, the target sequence can be replaced by changing only 20 internal nucleotides of two universal complementary oligo scaffolds (oligo scaffolds 23, 24 shown in Table S1). These oligos were designed to be complementary and when annealed will leave 10 pb overhangs that match the overhangs left on the backbone after USER treatment. Therefore it is necessary replace only the 20 bp sequence with the new target in a way that the two oligos sequences will be complementary (20 pb sequence on the forward oligo has to be complementary to the 20 bp sequence on the reverse oligo). The oligos were ordered without uracil making the method very cost efficient, and 10 μL of each oligo (100 μM concentrated) was then mixed with 10 μL of Neb Buffer4 and 70 μL of MilliQ Water (100 μL reaction in total). The reaction mix was incubated at 95 °C for 5 minutes and let it cool down slowly overnight. Once the two oligos were annealed, they were mixed with the amplified backbone, treated with USER enzymes and directly transformed into chemically competent cells. The self-killing plasmid containing the galk2.2 gRNA, xylA and LacZ were obtained with this method using primers 25–26, 27–28, 29–30 respectively, (Table S1) in just a single day of work. The second gRNA for multiplexing was inserted in pMAZ-SK::galK2 using primers 31 to 34 (Table S1). pMA7CR_2.0 and pMAZ-SK sequences are shown in Table S1.

### CRMAGE protocol

An overnight culture of MG1655::pMA7RCR_2.0 in LB with 100 μg/mL ampicillin (to maintain pMA7CR_2.0), 35 μg/mL chloramphenicol (to maintain pZS4Int-tetR if not integrated in the genome) was shaken at 37 °C. The following day 15 mL of LB with 100 μg/mL ampicillin, 35 μg/mL chloramphenicol was inoculated with 0.15 mL of the overnight culture in a 250 mL flask (starting OD between 0.02–0.03), incubated at 37 °C shaking and grown until an OD of 0.5–0.6 (about 1.5–2 hours). λ Red was induced by adding L-arabinose to a final concentration of 0.2%, followed by continued shaking at 37 °C for 10–15 minutes. The culture was immediately put in an ice-water bath and left to cool for at least 15–20 minutes. The culture was then pelleted by centrifugation at 6500 x *g* for 5 minutes at 4 °C, the supernatant was discarded and the pellet resuspended in 35 mL of ice-cold MilliQ water. This procedure was repeated two times more, where the pellet was first re-suspended in 15 mL and then in 1 mL of ice-cold MilliQ water. The 1 mL of cells were finally centrifuged at 6500–7000 x *g* for 5 minutes at 4 °C and the supernatant removed completely. Subsequently, cells were prepared for electroporation and re-suspended in 400–800 μL of ice-cold MilliQ water. The MAGE oligonucleotides together with the plasmid for negative selection were prepared in advance in an Eppendorf tube containing 0.5 μL of equimolar amounts of each oligo (10 pmol/μL of each) and 250 ng of the plasmid in MilliQ water. 50 μL of cells were added to the oligos/plasmid mix and electroporated at 1.8 kV in a 1 mm gap cuvette, and 950 μL of LB with 100 μg/mL ampicillin, 35 μg/mL chloramphenicol was added immediately after and the cells were transferred to a new tube (15 mL) and left to recover for 1 h at 37 °C with shaking. Kanamycin was added to reach a concentration of 50 μg/mL and the culture was incubated for additional 2 hours.

After 3 hours of incubation, aTetracycline (200 ng/mL) was added and the cells were grown for another 2 hours at 37 °C shaking. After this point the cells can either be plated on selective media or the plasmid for the negative selection can be rescued to start another round of MAGE. For the plasmid cure, the cells were washed twice with fresh LB and resuspended in LB with 100 μg/mL ampicillin, 35 μg/mL chloramphenicol, aTetracycline 200 ng/mL and 0.2% (w/v) of L-rhamnose. If the cell were left to grow ON, a smaller inoculum (10^3^–10^4^ dilution) was used. If the culture was prepared for an immediate subsequent round of CRMAGE, the it was diluted to an OD that allows at least 2–3 h of growth before reaching OD:0.5–0.6, which is necessary to start the following round.

### CRMAGE Web-Tool

The CRMAGE web-based design tool is available at http://staff.biosustain.dtu.dk/laeb/crmage/and follows three individual steps as described here: Step 1: Enter the wild type DNA sequence. Simply copy/paste or manually enter the sequence. Step 2: Select the base to mutate. Here the user is presented with a numbered and spaced version of the sequence entered in step 1 and the user can then click the exact base that should be mutated and can select into which base it should be mutated. Step 3: CRMAGE mutation oligo and gRNA. In this step the user can select a desired CRMAGE mutation oligo size. The size of the MAGE oligo determines which gRNA sites can be used since the program sets that as constraint region from which it samples the possible gRNAs to use as “negative selection marker”. The user should then select the correct reading frame. Then a list of potential gRNA sites are shown and the user can pick silent mutations with the intent to destroy the indicated gRNA PAM site. Be aware that all silent mutations are shown even if they do not destroy the gRNA PAM site. It is up to the user to ensure that the PAM site is destroyed. One or two mutation boxes may be shown next to a gRNA target. This is dependent on whether one or two codons cover the PAM site. At the very end of step 3, the mutation oligo sequence is presented and can be copy/pasted for synthesis.

## Additional Information

**How to cite this article**: Ronda, C. *et al.* CRMAGE: CRISPR Optimized MAGE Recombineering. *Sci. Rep.* **6**, 19452; doi: 10.1038/srep19452 (2016).

## Supplementary Material

Supplementary Information

## Figures and Tables

**Table 1:**
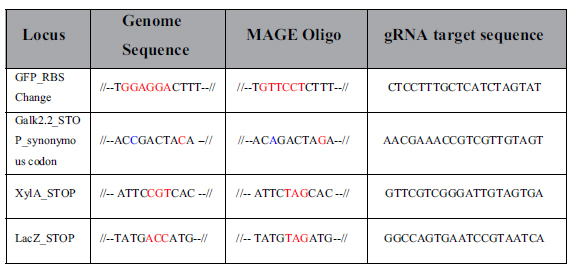
Oligos used for the CRMAGE experiments.

Target sites are marked with red. Synonymous mutations are in blue.
