# Supplementary material for: CRMAGE: CRISPR Optimized MAGE Recombineering
Source: Sci Rep. 2016 Jan 22;6:19452. doi: 10.1038/srep19452 (PMC4726160; doi:10.1038/srep19452)
Supplement: Supplementary Information [file srep19452-s1.doc]

CRMAGE: CRISPR Optimized MAGE Recombineering

Carlotta Ronda, Lasse Ebdrup Pedersen, Morten O.A. Sommer, Alex Toftgaard Nielsen
The Novo Nordisk Foundation Center for Biosustainability, Technical University of Denmark, Kogle Allé 6, 2970 Hørsholm, Denmark

Table S1: Primers and oligos used in the study

|  | **Primer** |  | **Source** |
| --- | --- | --- | --- |
| 1 | recX_fwd_kpnI | ATCGAGGTACCATGACAGAATCAACATCCCGTC | This study |
| 2 | recX_rev_BamHI | TCGTAGGATCCATCAGTCGGCAAAATTTCGCCA | This study |
| 3 | pMA7_fwd | AAGCGGGUTTTTTTATGACAAACTCTTTTGTTTATT | This study |
| 4 | pMA7_rev | ATAGGGAATAGGGAGUAGAAACGCAA | This study |
| 5 | fwd_insert_recX | ACTCCCTATTCCCTAUCAGTGATAGAGATTGACAT | This study |
| 6 | rev_insert_recX | ACCCGCTUGCGCGGGCTTTTTCACATTGATGCCTCTAGCACGC | This study |
| 7 | pMA7CR_2.0_fwd | ACTGAGA/ideoxyU/CCCATGGTACGCGTG | This study |
| 8 | pMA7CR_2.0_rev | ATCTCCTTC/ideoxyU/caGTCGGCAAAATTTCG | This study |
| 9 | c9_PMA7CR_2.0 fwd | AGAAGGAGA/ideoxyU/ATACATGGATAAGAAATACT | This study |
| 10 | c9_PMA7CR_2.0 rev | ATCTCAG/ideoxyU /CACCTCCTAGCTGACTCA | This study |
| 11 | trancrRNA_Coli_fwd | ACACCGAC/ideoxyU/AGCGAAAAAACC | This study |
| 12 | trancrRNA_Coli_rev | ACGCTGCT/ideoxyU/TTGACGGCTA | This study |
| 13 | pCOLAtran_rev | AGTCGGTG/ideoxyU/GCGCAACGCAATTAATGTA | This study |
| 14 | pCOLAtran_fwd | AAGCAGCG/IDEOXYU/ATATACCATGGGCAGCAGC | This study |
| 15 | Part2_selfkilling | TTAATTAACACCGACTAGCGAAAAAACCCCGCCGAAGCGGGGTTTTTTGCGAAAAAAAGCACCGACTCGGTGCCACTTTTTCAAGTTGATAACGGACTAGCCTTATTTTAACTTGCTATGCTGTTTTGAATGGTTCCGCTAGCACTGTACCTAGGACTGAGCTAGCCGTCAAAAGCAGCGTCCTCAGG | This study |
| 16 | Part1_selfkilling | CCATGGCCACAATTCAGCAAATTGTGAACATCATCACGTTCATCTTTCCCTGGTTGCCAATGGCCCATTTTCCTGTCAGTAACGAGAAGGTCGCGAATTCAGGCGCTTTTTAGACTGGTCGTGTTTTAGAGCTATGCTGTTTTGAATGGTCCCAAAACCCGTTCCGTGTAGACAGTTCGCTCCAAGCTGTTTTAGAGCTATGCTGTTTTGAATGGTCCCAAAACGCCATGTTTCAGAAACAACTCTGGCGCATCGTTTTAGAGCTATGCTGTTTTGAATGGTCCCAAAACAAAAAAAAACCCCGCCCCTGACAGGGCGGGGTTTTTTTTTTAATTAA | This study |
| 17 | pCOLAforgrna_fwd | ACCGGTA/ideoxyU/CCTAGGCTGCTGCCACCG | This study |
| 18 | pCOLAforgrna_rev | ACCAGAC/IDEOXY U/TTAATTAAAAAAAAAACCCCGCCCTGTCA | This study |
| 19 | pCOLA_gRNA_fwd | AGTCTGG/ideoxyU/TATAACCTGAGGTCCCTATCAGTGATAGAGA | This study |
| 20 | pCOLA_gRNA_rev | ATACCGG/ideoxyU/TTCGACTTAAGCATTATGCGG | This study |
| 21 | new universal backbone fwd primer | AGCTAGAAA/ideoxyU/AGCAAGTTAAAATAAGGC | This study |
| 22 | New rev back bone primer | AGTATCTC/ideoxyU/ATCACTGATAGGGATGTCA | This study |
| 23 | variable region fwd | GAGCAC (20N) GTTTTAGAGCTAGAAAT | This study |
| 24 | Variable region rev | CTAAAAC(20N)GTGCTCAGTATCTCT | This study |
| 25 | Variable region galk2 fwd | **GAGCAC**AACGAAACCGTCGTTGTAGT**GTTTTAGAGCTAGAAAT** | This study |
| 26 | Variable region galk2 rev | **CTAAAAC**ACTACAACGACGGTTTCGTT**GTGCTCAGTATCTCT** | This study |
| 27 | Variable region xylA fwd | **GAGCAC**GTTCGTCGGGATTGTAGTGA**GTTTTAGAGCTAGAAAT** | This study |
| 28 | Variable region xylA rev | **CTAAAAC**TCACTACAATCCCGACGAAC**GTGCTCAGTATCTCT** | This study |
| 29 | Variable region LacZ fwd | **GAGCAC**GGCCAGTGAATCCGTAATCA**GTTTTAGAGCTAGAAAT** | This study |
| 30 | Variable region LacZ rev | **CTAAAAC**TGATTACGGATTCACTGGCC**GTGCTCAGTATCTCT** | This study |
| 31 | 2_sgRNA_fwd | ACCGGTATTCCCTATCAGTGATAGAGAT | This study |
| 32 | 2_sgRNA_rv | AGCAGCCTAGGAAAAAAGCACCGACTCGG | This Study |
| 33 | 2_sgRNAbb_fwd | AGGCTGCTGCCACCGCTGA | This Study |
| 34 | 2_sgRNAbb_rev | ATACCGGTAAAAAAGCACCGACTCGGTGCCA | This Study |
| 35 | Oligo for GFP negative (weak RBS) | GACAACTCCAGTGAAAAGTTCTTCTCCTTTGCTCATCTAGTATTGGAGGACTTTAATCTCTAGTAGCTAGCACTGTACCTAGGACTGAGC | This Study |
|  | **CRMAGE oligos** |  |  |
|  | GFP_RBS CHANGE | GACAACTCCAGTGAAAAGTTCTTCTCCTTTGCTCATCTAGTATTGTTCCTCTTTAATCTCTAGTAGCTAGCACTGTACCTAGGACTGAGC | This study |
|  | Galk2.2_STOP_synonymous codon | GGCCGCGTGAATTTGATTGGTGAACACACAGACTAGAACGACGGTTTCGTTCTGCCCTGCGCGATTGATT | This study |
|  | XylA_STOP | AGGCTCAAAATCCTCAAACCCGTTAGCATTCTAGCACTACAATCCCGACGAACTGGTGTTGGGTAAGCGT | This study |
|  | LacZ_STOP | CGGATAACAATTTCACACAGGAAACAGCTATGTAGATGATTACGGATTCACTGGCCGTCGTTTTACAACG | This study |
|  | **Synthetic gRNA** |  |  |
|  | Promoter (pLtet) | TCCCTATCAGTGATAGAGATTGACATCCCTATCAGTGATAGAGATACTGAGCAC | This study ^56^ |
|  | Scaffold | GTTTTAGAGCTAGAAATAGCAAGTTAAAATAAGGCTAGTCCGTTATCAACTTGAAAAAGTGGCACCGAGTCGGTGCTTTTTT | 37,38 |
|  | GFP4 target | CTCCTTTGCTCATCTAGTAT | This study |
|  | Galk 2 target | AACGAAACCGTCGTTGTAGT | This study |
|  | **crRNA Array** |  |  |
|  | pRham | CCACAATTCAGCAAATTGTGAACATCATCACGTTCATCTTTCCCTGGTTGCCAATGGCCCATTTTCCTGTCAGTAACGAGAAGGTCGCGAATTCAGGCGCTTTTTAGACTGGTCGT | This study |
|  | Repeat | GTTTTAGAGCTATGCTGTTTTGAATGGTCCCAAAAC | This study |
|  | Target Ori | CCGTTCCGTGTAGACAGTTCGCTCCAAGCT | This study |
|  | Target Kanamycin | GCCATGTTTCAGAAACAACTCTGGCGCATC | This study |
|  | Synthetic Terminator for crRNA array(BBa_b1006) | AAAAAAAAACCCCGCCCCTGACAGGGCGGGGTTTTTTTT | This study |
|  | trascrRNA |  |  |
|  | trascrRNA sequence | GGAACCATTCAAAACAGCATAGCAAGTTAAAATAAGGCTAGTCCGTTATCAACTTGAAAAAGTGGCACCGAGTCGGTGCTTTTTTT | This study,^42^ |
|  | Promoter for trascrRNA (BBa_J23100pr) | TTGACGGCTAGCTCAGTCCTAGGTACAGTGCTAGC | This study |
|  | Terminator for trascrRNA (BBab_1002) | CGCAAAAAACCCCGCTTCGGCGGGGTTTTTTCGC | This study |

**Figure S1:**

**
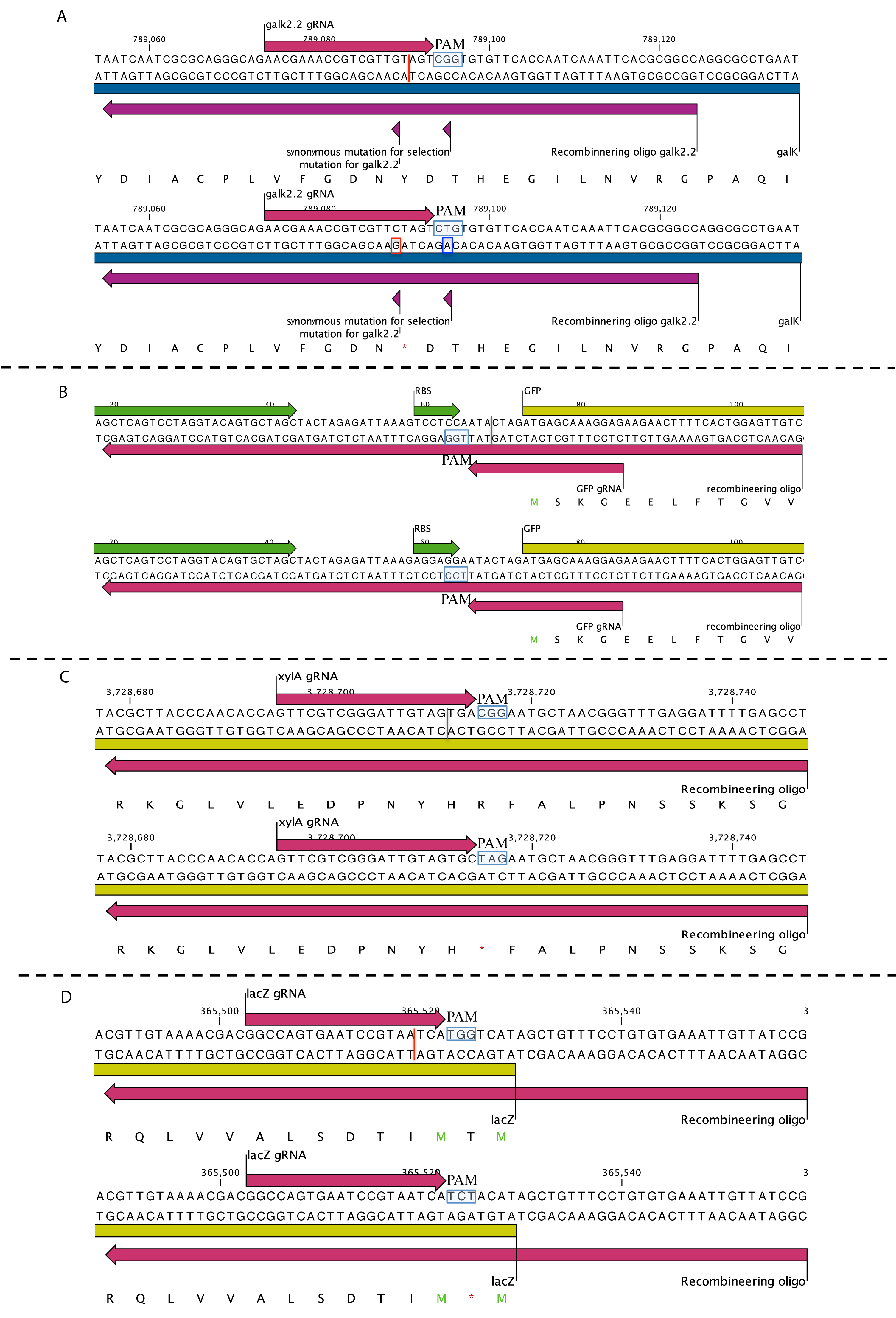
**

Figure. S1: Overview of the target loci. On top is the site before the modification and below is after it has been modified with recombineering and positively selected with CRISPR for the following targets (A) *galK*, (B) GFP, (C) *xylA*, and (D) *lacZ*. The coding sequences are shown in blue and yellow, the long pink oligos on the minus strand are the ones used for the recombineering to introduce the targeted mutations. The short 20 bp regions annotated as pink represent the gRNA target sequences. The light blue rectangles indicate the PAM sites used as discriminant for negative selection. The red line on the top sequences (wild type) displays the Cas9 restriction site. Red stars in the translational frame represent stop codons. (A) The small blue rectangle shows the synonymous mutation used to change the PAM for the negative selection and the red rectangle underlines the targeted mutation that causes a stop of transcription.
